# Effects of the 5-Hydroxytryptamine 3 Receptor Antagonist Palonosetron on Hemostasis: An In Vitro Study Using Thromboelastography

**DOI:** 10.3390/medicina61040682

**Published:** 2025-04-08

**Authors:** Hyun-Jung Shin, Bon-Wook Koo, Ji-Na Kim, Ji-In Park, Hyo-Seok Na

**Affiliations:** 1 Department of Anesthesiology and Pain Medicine, Seoul National University Bundang Hospital, Seongnam 13620, Republic of Koreaanejina84@gmail.com (J.-N.K.);; 2 Department of Anesthesiology and Pain Medicine, Seoul National University College of Medicine, Seoul 03080, Republic of Korea

**Keywords:** palonosetron, platelet function, thromboelastography, hemostasis, 5-HT_3_ receptor antagonist, serotonin

## Abstract

*Background and Objectives*: Serotonin modulates platelet aggregation and secretion, but its role in hemostasis remains controversial. This study hypothesized that the 5-HT_3_ receptor antagonist palonosetron may inhibit platelet function and aimed to evaluate its effects on blood coagulation using thromboelastography (TEG). *Materials and Methods*: Blood samples from 11 healthy volunteers were treated with palonosetron at concentrations of 25, 250, and 2500 ng/mL. Untreated samples served as controls. Coagulation parameters were assessed using global hemostasis (citrated kaolin, citrated rapid TEG, citrated kaolin with heparinase, and citrated functional fibrinogen) and PlateletMapping (adenosine diphosphate [ADP], arachidonic acid, and others) assays. *Results*: In the global hemostasis assay, maximum amplitude values, reflecting clot strength, decreased with increasing palonosetron concentrations in all tests, including citrated kaolin (*p* = 0.031), citrated rapid TEG (*p* = 0.001), citrated kaolin with heparinase (*p* = 0.033), and citrated functional fibrinogen (*p* = 0.011). The PlateletMapping assay showed significant reductions in ADP-induced platelet aggregation (*p* = 0.001), with the largest inhibition observed at 2500 ng/mL (*p* = 0.007). Despite these changes, all values remained within normal reference ranges. *Conclusions*: Palonosetron induces hypocoagulable trends in vitro by inhibiting platelet function and fibrinogen-mediated clot strength. However, these changes are unlikely to result in clinically significant hemostatic impairment when used within therapeutic doses. Further research is warranted to confirm these findings and explore their clinical relevance.

## 1. Introduction

Several surgical procedures are associated with a risk of significant bleeding or thrombosis, which can lead to adverse outcomes such as organ dysfunction, prolonged hospitalization, and even death [1]. Blood coagulation may change perioperatively due to various factors, including drugs, fluids, tissue injury, inflammation, and other numerous unpredictable factors [2,3,4]. These changes involve the activation of clotting factors, the inhibition of anticoagulant pathways, and the consumption of platelets and coagulation factors.

Platelets play a crucial role in hemostasis by forming aggregates at the site of vessel injury and releasing various factors that promote coagulation and vascular repair [5]. However, the regulation of platelet function is complex and involves multiple signaling pathways [5]. Of those, the role of 5-hydroxytryptamine (5-HT) or serotonin on platelet function has been continuously studied [6,7,8]. However, its exact role in hemostasis and thrombosis remains controversial, even though 5-HT is a potent vasoconstrictor and stimulator that can modulate platelet aggregation and secretion [9].

Recently, the expression of 5-HT_3_ receptors on the platelet surface has been observed [10]. The 5-HT_3_ receptor antagonists, such as ondansetron, palonosetron, and ramosetron, are used widely for managing postoperative or chemotherapy-induced nausea and vomiting [11] and potentially may affect the hemostasis through their interactions with 5-HT_3_ receptors on platelets [12]. Although 5-HT_3_ receptor antagonists showed an anti-aggregation effect on platelets [12] and lowered the incidence of deep vein thrombosis [13], the exact mechanism remains unclear.

In this study, we aimed to elucidate the impact of 5-HT_3_ receptor antagonists on blood coagulation and platelet function using thromboelastography (TEG). Our primary focus was to investigate whether palonosetron exerts a direct inhibitory effect on hemostasis by assessing changes in TEG parameters in vitro. Additionally, given that serotonin influences multiple coagulation pathways, we sought to distinguish between direct receptor-mediated effects and potential indirect mechanisms such as interactions with adenosine diphosphate (ADP) receptor-related pathways. Specifically, we sought to assess the coagulation status and platelet function, according to the concentration of palonosetron, through TEG.

## 2. Materials and Methods

Ethics approvals were obtained from the Institutional Review Board of Seoul National University Bundang Hospital (approval granted on 14 August 2020; B-2008/631-304) and were registered in the clinical trial database (https://clinicaltrials.gov, NCT04507711, accessed on 9 August 2020). This study was carried out by collecting blood from healthy volunteers in accordance with the Declaration of Helsinki. Written informed consent was obtained from healthy adult participants under the age of 50 who were not taking any agents known to inhibit hemostasis, such as antiplatelet, anticoagulant, and nonsteroidal anti-inflammatory drugs. Initially, a total of 12 healthy volunteers provided informed consent to participate in the present study. After excepting one participant who took nonsteroidal anti-inflammatory drugs before blood sampling, data were collected and analyzed from eleven participants—six female and five male volunteers (age range: 30–45 years)—who had no comorbidities and no current medication use.

### 2.1. Global Hemostasis Assay

To evaluate blood coagulation status, whole blood that was drawn from volunteers was placed into a citrate-containing polypropylene tube (Vacutainer, Becton Dickinson, Plymouth, UK) immediately. The collected blood samples were divided into four bottles, each containing 2 mL of blood.

We selected the reference range for palonosetron concentrations (25, 250, and 2500 ng/mL) based on previously published pharmacokinetic data, which reported peak plasma concentrations ranging from 13.0 ± 20.1 ng/mL to 336 ± 940 ng/mL, following standard dosing [14]. To ensure adequate coverage of both therapeutic and potential supratherapeutic levels, we adopted a wider range. While the upper limit of 2500 ng/mL exceeds typical therapeutic levels, this broader range was chosen to accommodate potential interindividual variability and to enhance the sensitivity of the assay. To generate the final sample concentrations, 1, 10, and 100 μL of palonosetron (Aloxi, 0.075 mg/1.5 mL, Pierre-Fabre Medicament Production, Boulogne Billancourt, France) was added to three bottles, respectively, after the same volume of blood was discarded. In the fourth bottle, no drugs were added to the blood sample, and this sample was used as the baseline value (0 ng/mL, control).

All analyses were performed using a thromboelastography machine (TEG 6s, Hemonetics, Boston, MA, USA), according to the manufacturer’s recommendations. The global hemostasis assay was performed automatically after inserting a cartridge containing reagents for the following four tests: a kaolin activation test (citrated kaolin, CK), which primarily evaluates the intrinsic pathway; a kaolin and tissue factor activation test (citrated rapid TEG, CRT), which evaluates both intrinsic and extrinsic pathways; a kaolin and heparinase activation test (citrated kaolin with heparinase, CKH), which neutralizes the effect of heparin in the test sample; and a functional fibrinogen test (citrated functional fibrinogen, CFF), which isolates fibrinogen’s contribution to clot strength by using a potent glycoprotein IIb/IIIa platelet inhibitor to suppress platelet activity. The following four parameters were obtained from each test: reaction time (R, min), clot formation kinetics (K, min), maximum amplitude (MA, mm), and alpha angle (α, degrees). The MA values from CK, CKH, and CRT primarily reflect platelet function, whereas the MA value of CFF reflects fibrinogen function [15].

### 2.2. PlateletMapping Assay

For the PlateletMapping assay, whole blood was collected in a heparin-containing tube (Vacutainer, Becton Dickinson, Franklin Lakes, NJ, USA), and palonosetron was prepared in those tubes in the same manner as for the global hemostasis test, except at a concentration of 25 ng/mL, which has shown no significant effect on blood coagulation through global hemostasis assays.

Four measurements were included in the PlateletMapping assay: a kaolin activation with heparinase test (HKH), an ADP test, an arachidonic acid (AA) activation test, and an activator F test (ActF), which uses abciximab (GP IIb/IIIa inhibitor) to inhibit platelet function and measure viscoelasticity caused by fibrin network formation triggered by reptilase and factor XIII.

### 2.3. Outcomes

Assuming that the major focus would be the effect of 5-HT_3_ receptor antagonists on platelets, the change in MA values according to the drug concentrations in the global hemostasis assay was designated as the primary outcome. The other parameters of the global hemostasis assay and the results of PlateletMapping assays were included in the secondary outcome.

### 2.4. Sample Size

In a study which was performed with healthy volunteers [16], the value of MA of CRT was 63 (2.5) mm. Assuming a 5% difference in the MA of CRT from the baseline values and aiming for a power of 80% at the 5% significance level, it was estimated that 12 participants would be required for our study, anticipating a dropout rate of 30%.

### 2.5. Statistical Analysis

The obtained data were expressed as the mean (standard deviation). Data were analyzed using SPSS for Windows (ver. 27; IBM Corp., Armonk, NY, USA). The normality test was performed using the Shapiro–Wilk test. Repeated measures analysis of variance was used to determine whether there was a change in mean TEG 6s values according to the drug concentrations, and a *p*-value of < 0.05 was treated as indicating statistical significance. Thereafter, detailed differences in each TEG 6s value among the concentrations were analyzed using a paired *t*-test as a post hoc analysis. For this multiple comparison adjusted by Bonferroni correction, a *p*-value < 0.017 (0.05/3, in the global hemostasis assay) or <0.025 (0.05/2, in the PlateletMapping assay) was considered statistically significant appropriately.

## 3. Results

### 3.1. Global Hemostasis Assays

Table 1 shows the results of global hemostasis assays. In the present study, the values of MA of CK (*p* = 0.031), CRT (*p* = 0.001), CKH (*p* = 0.033), and CFF (*p* = 0.011) tests were decreased significantly as the concentration of palonosetron increased. When we performed the post hoc comparisons, using a paired *t*-test, between 0 ng/mL (baseline) and each concentration, there were significant decreases in the MA values of CRT in the 250 ng/mL (*p* = 0.002) and 2500 ng/mL (*p* = 0.002) samples and in the MA values of CFF in the 250 ng/mL (*p* = 0.003) and 2500 ng/mL (*p* = 0.006) samples.

The R (*p* = 0.002) and K (*p* = 0.015) values in the CRT test showed concentration-dependent prolongation. When performing post hoc tests, significant differences were observed between the baseline and the 2500 ng/mL samples (*p* = 0.011) in the R values of the CRT test. Furthermore, the value of K of CRT was prolonged in the 250 ng/mL (*p* = 0.005) and 2500 ng/mL (*p* = 0.008) samples of palonosetron. However, all values of the tests were within the normal reference range regardless of the drug concentrations.

### 3.2. PlateletMapping Assay

The results of the PlateletMapping assay are shown in Table 2. Among the four parameters (HKH, ActF, ADP, and AA), significant changes were presented in the ADP test. The MA of ADP was decreased with a rising concentration (*p* = 0.001). In particular, the lowest value of MA was observed in the 2500 ng/mL sample compared with the baseline value in post hoc analysis (*p* = 0.007). As the concentration of palonosetron increased, the degree of inhibition increased and aggregation decreased (*p* = 0.004, respectively). Furthermore, most large changes were observed in the 2500 ng/mL sample from the baseline values in both the degree of inhibition and aggregation (*p* = 0.004, respectively).

## 4. Discussion

In this study, the effects of the 5-HT_3_ receptor antagonist palonosetron on hemostasis were evaluated using both a global hemostasis assay and the PlateletMapping assay. The results demonstrated significant reductions in the MA values across multiple tests in the global hemostasis assay, indicating a dose-dependent hypocoagulable effect as palonosetron concentrations increased. Despite these changes, all parameters remained within the normal reference ranges, suggesting minimal clinical significance when palonosetron is used within therapeutic doses. The PlateletMapping assay confirmed that the platelet function was inhibited by palonosetron in the ADP activation test.

Serotonin has been known to have procoagulant properties in hemostasis via activating the 5-HT_2_ receptors found on the platelet surface [17]. Galan et al. [18] identified that serotonin enhanced platelet thrombus formation and increased fibrin deposition on the damaged vascular surface.

Selective serotonin re-uptake inhibitors, which are used as an anti-depressant, showed an anticoagulant effect in various clinical trials [17,19]. Ondansetron, a 5-HT_3_ receptor antagonist, attenuated platelet aggregation in a dose-dependent manner, and, interestingly, inositol triphosphate (IP3) signaling and the mitogen-activated protein (MAP) kinase pathway were involved in addition to the 5-HT_3_ receptor-dependent pathway [12]. Clinically, the risk of hospital-acquired venous thromboembolism was reduced when ondansetron was used, as observed in a retrospective cohort study [13]. To the best of our knowledge, no clinical studies to date have specifically examined the effects of other 5-HT_3_ receptor antagonists, with the exception of ondansetron, on platelets. In the present study, palonosetron was selected due to its longer half-life (approximately 40 h) and stronger binding affinity for the 5-HT_3_ receptor compared to ondansetron [20].

In this study, a TEG 6s analyzer was used to evaluate the antiplatelet activity of palonosetron. Global hemostasis assays can assess the overall blood coagulation status, including platelet contribution (value of MA), and the PlateletMapping assay can provide information related to platelet function [15]. As expected, we were able to identify reduced MA values and observe some changes in the R and K of the CRT in the global hemostasis assay. The MA values of CK, CRT, and CKH reflect platelet activity, while the MA value of CFF specifically evaluates fibrinogen’s role in clot strength. The observed reductions in MA of CFF suggest that palonosetron may interfere with fibrinogen aggregation or polymerization, possibly through diminished thrombin activity or other indirect pathways. Additionally, prolongations in the R and K values in the CRT test indicate delayed thrombin generation, which could impair fibrin formation and overall clot stability. Although there is no direct evidence linking palonosetron to defects in fibrinogen aggregation, these results highlight the need for further investigation into its potential effects on coagulation, especially regarding its possible influence on fibrinogen function via platelet or thrombin-mediated mechanisms.

The PlateletMapping assay confirmed that palonosetron inhibits platelet aggregation in the ADP activation test, consistent with the reductions in platelet-driven MA values in the global hemostasis assay. When considering that the ADP plays a crucial role in platelet aggregation through P2Y12 and P2Y1 receptors [21], palonosetron may impact the platelet aggregation triggered by these receptors' activation. This hypothesis can be supported by a study that suggested the involvement of IP3 and MAP kinase, which are intracellular pathways triggered by the interaction of ADP with P2Y12 and P2Y1 receptors [22], in the anti-aggregation effect of ondansetron on platelets [12]. The present study identified the effect of palonosetron on blood coagulation and platelet function, showing inhibition, but the exact mechanism of the participation of the 5-HT_3_ receptor activation cascades in the hemostasis could not be explained. In considering the inhibition of the ADP-induced pathway and the lack of effect on the AA-activated pathway, it suggests that the influence may be on the platelet function activation cascade involving ADP rather than AA. Further study is needed to overcome this limitation.

Although the findings demonstrate that palonosetron induces hypocoagulable changes in vitro, the clinical relevance of these changes is uncertain. Importantly, all global hemostasis assay parameters, including the prolonged R and K values and reduced MA values, remained within normal reference ranges, even at palonosetron concentrations significantly exceeding therapeutic levels. This indicates that palonosetron, when used as a single agent within the therapeutic range, is unlikely to cause clinically significant hemostatic impairment. However, the perioperative period is characterized by complex interactions among various drugs, physiological stressors, and immune responses. In patients with pre-existing coagulopathies or those exposed to multiple anticoagulant agents, the hypocoagulable effects of palonosetron may require closer monitoring. Patients undergoing high-risk surgeries, such as major orthopedic, cardiovascular, neurosurgical, or hepatic procedures, may be particularly susceptible to subtle coagulation changes. In these populations, even small alterations in clot formation dynamics could contribute to an increased risk of perioperative bleeding. Conversely, in patients with prothrombotic conditions, the potential for palonosetron to reduce platelet aggregation might offer a protective effect against thrombotic complications. Future studies focusing on these patient populations will be essential to determine whether specific precautions should be taken in clinical practice, including individualized risk assessments or modified perioperative management strategies.

Several limitations should be considered when interpreting the present results. Firstly, our study was conducted using an in vitro model, which does not fully account for the physiological compensatory mechanisms present in vivo. Factors such as vascular interactions, endothelial function, fibrinolysis, and the dynamic interplay of coagulation regulators may influence the actual hemostatic effects of palonosetron in a clinical setting. In vivo conditions also involve the continuous renewal of platelets and clotting factors, which may mitigate or amplify the drug’s impact on coagulation. Future research incorporating in vivo models or clinical trials will be necessary to provide a more comprehensive understanding of how palonosetron interacts with hemostasis in real-world patient populations. Secondly, the signaling process, on a molecular level, could not be explained in this study. Although palonosetron attenuated platelet function, resulting in a hypocoagulable change in hemostasis, more research is needed to confirm the involvement of 5-HT_3_ receptors on the platelet surface. Thirdly, the actual intraoperative hemorrhagic condition could not be demonstrated via this experimental study from initial bleeding to fibrinolysis step. It might be very complex and difficult to construct a suitable model to evaluate the effect of 5-HT_3_ receptor antagonists. Further well-planned trials are required to confirm our results clinically. Fourthly, we did not investigate platelet counts, which have a significant relationship with the MA values of CKH and CRT [15]. Nevertheless, the trends toward hypocoagulable status could be observed according to the elevation of the drug’s palonosetron concentrations. Fifth, the small sample size of 11 donors presents a limitation in terms of effect size estimation and statistical power. While power calculations were performed, a larger sample size could yield more robust conclusions and enhance generalizability. The limited sample size may also contribute to variability in the results and reduce the ability to detect smaller but potentially meaningful differences. Future studies with larger participant groups will be essential to validate and expand upon these findings. A final limitation of this study is the use of a palonosetron concentration of 2500 ng/mL, which exceeds the typical therapeutic range. This concentration was included to explore potential dose-dependent effects and to provide a broader understanding of its impact on platelet function and coagulation parameters. However, since these in vitro conditions may not fully replicate clinical scenarios, caution is advised when extrapolating these findings to actual patient care. Future research involving in vivo studies and clinically relevant dosing ranges will be essential to confirm the clinical significance of these observations.

## 5. Conclusions

In conclusion, this study demonstrated that palonosetron induces hypocoagulable trends in vitro, as evidenced by reduced MA values in the global hemostasis assay and inhibition of platelet aggregation in the PlateletMapping assay. These effects likely reflect impairments in platelet function and fibrinogen-mediated clot strength, potentially mediated by delayed thrombin generation and interference with ADP-induced platelet aggregation. Despite these findings, the changes remained within normal reference ranges, indicating that palonosetron is unlikely to cause clinically significant hemostatic impairment when used within therapeutic doses. Further research, particularly in clinical perioperative settings, is needed to elucidate the molecular mechanisms and confirm these findings.

## Figures and Tables

**Table 1:** Global hemostasis assay according to the concentration of palonosetron.

	Reference Values	Palonosetron Concentration (*n* = 11)				*p*-Value
		0 ng/mL	25 ng/mL	250 ng/mL	2500 ng/mL	
CK						
R (min)	4.6–9.1	6.5 (0.8)	6.4 (0.9)	6.5 (0.8)	6.6 (0.8)	0.341
K (min)	0.8–2.1	1.6 (0.3)	1.5 (0.3)	1.4 (0.4)	1.6 (0.5)	0.551
MA (mm)	52–69	59.9 (3.5)	58.0 (4.5)	58.7 (3.7)	56.8 (4.8)	0.031
CRT						
R (min)	0.3–1.1	0.5 (0.1)	0.5 (0.1)	0.4 (0.1)	0.6 (0.2) *	0.002
K (min)	0.8–2.7	1.5 (0.4)	1.6 (0.5)	1.7 (0.5) *	1.9 (0.5) *	0.015
MA (mm)	52–70	61.0 (4.4)	59.3 (4.7)	58.5 (4.5) *	56.6 (5.2) *	0.001
CKH						
R (min)	4.3–8.3	6.4 (0.8)	6.5 (0.9)	6.5 (0.9)	6.6 (0.9)	0.151
K (min)	0.8–1.9	1.6 (0.4)	1.5 (0.4)	1.4 (0.3)	1.6 (0.5)	0.599
MA (mm)	52–69	60.1 (3.5)	58.9 (3.2)	58.3 (4.4)	57.4 (4.7)	0.033
CFF						
MA (mm)	13–30	19.8 (0.3)	19.4 (0.3)	19.0 (0.4) *	16.8 (0.5) *	0.011

Data are expressed as mean (standard deviation). *p*-values result from the repeated measured analysis of variance in each parameter. * *p*-value < 0.017 in comparison between 0 ng/mL and each concentration by a paired *t*-test as a post hoc analysis. CK, citrated kaolin; CRT, citrated rapid thromboelastography; CKH, citrated kaolin with heparinase; CFF, citrated functional fibrinogen; R, reaction time; K, K-time; MA, maximum amplitude.

**Table 2:** PlateletMapping assay according to the concentration of palonosetron.

	Reference Values	Palonosetron Concentration (*n* = 11)			*p*-Value
		0 ng/mL	250 ng/mL	2500 ng/mL	
HKH					
R (min)	4.2–9.8	11.2 (7.0)	8.3 (3.8)	9.8 (3.6)	0.379
K (min)	1.0–2.9	3.8 (2.0)	3.4 (1.8)	4.5 (2.6)	0.232
MA (mm)	53–68	50.7 (7.6)	50.7 (8.2)	47.3 (9.7)	0.130
ActF					
MA (mm)	2–19	6.4 (4.6)	7.1 (5.4)	4.7 (2.6)	0.100
ADP					
MA (mm)	45–69	42.8 (5.2)	34.2 (10.3)	22.8 (4.5) *	0.001
Inhibition (%)		17.5 (2.6)	37.3 (19.2)	56.4 (16.3) *	0.004
Aggregation (%)		82.5 (2.6)	62.7 (19.2)	43.6 (16.3) *	0.004
AA					
MA (mm)	51–71	41.1 (13.6)	36.2 (10.0)	30.8 (5.8)	0.201
Inhibition (%)		26.7 (28.5)	34.5 (20.3)	37.0 (22.0)	0.470
Aggregation (%)		75.3 (28.5)	65.5 (20.3)	62.9 (22.0)	0.470

Data are expressed as mean (standard deviation). *p*-values result from the repeated measured analysis of variance in each parameter. * *p*-value < 0.025 in comparison between 0 ng/mL and each concentration by a paired *t*-test as a post hoc analysis. HKH, a kaolin activation with heparinase; ActF, activator F; ADP, adenosine diphosphate; AA, arachidonic acid; R, reaction time; K, K-time; MA, maximum amplitude.

## Data Availability

The data presented in this study are available on request from the corresponding author.

## Abbreviations

The following abbreviations are used in this manuscript:

TEG	Thromboelastography
CK	Citrated kaolin
CRT	Citrated rapid TEG
CKH	Citrated kaolin with heparinase
CFF	Citrated functional fibrinogen
ADP	Adenosine diphosphate
AA	Arachidonic acid
R	Reaction time
K	Clot formation kinetics
MA	Maximum amplitude
α	Alpha angle
HKH	Kaolin activation with heparinase test
ActF	Activator F test
5-HT	5-hydroxytryptamine
